# Comparison of immune responses to SARS-CoV-2 spike following Omicron infection or Omicron BA.4/5 vaccination in kidney transplant recipients

**DOI:** 10.3389/fimmu.2024.1476294

**Published:** 2025-01-14

**Authors:** Inga Tometten, Tobias Brandt, Maike Schlotz, Ricarda Stumpf, Sinje Landmann, Marta Kantauskaite, Joshua Lamberti, Jonas Hillebrandt, Lisa Müller, Margarethe Kittel, Katrin Ivens, Henning Gruell, Anja Voges, Heiner Schaal, Nadine Lübke, Eva Königshausen, Lars Christian Rump, Florian Klein, Johannes Stegbauer, Jörg Timm

**Affiliations:** ^1^ Institute of Virology, Medical Faculty, University Hospital Düsseldorf, Heinrich-Heine-University Düsseldorf, Düsseldorf, Germany; ^2^ Department of Nephrology, Medical Faculty, University Hospital Düsseldorf, Heinrich-Heine-University Düsseldorf, Düsseldorf, Germany; ^3^ Laboratory of Experimental Immunology, Institute of Virology, Faculty of Medicine and University Hospital Cologne, University of Cologne, Cologne, Germany; ^4^ KfH Kuratorium für Dialyse und Nierentransplantation e.V., Düsseldorf, Germany; ^5^ Center for Molecular Medicine Cologne (CMMC), University of Cologne, Cologne, Germany; ^6^ German Center for Infection Research (DZIF), partner site Bonn-Cologne, Cologne, Germany

**Keywords:** SARS-CoV-2, Omicron BA.4/5, Omicron XBB.1.5, bivalent vaccination, kidney transplant recipients

## Abstract

**Background:**

The emergence of novel SARS-CoV-2 variants challenges immunity, particularly among immunocompromised kidney transplant recipients (KTRs). To address this, vaccines have been adjusted to circulating variants. Despite intensive vaccination efforts, SARS-CoV-2 infections surged among KTRs during the Omicron wave, enabling a direct comparison of variant-specific immunity following-vaccination against Omicron BA.4/5 or Omicron infection in KTRs.

**Methods:**

98 SARS-CoV-2 naïve KTRs who had received four vaccine doses were studied. Before and after a 5th antigen exposure, either via the bivalent vaccine composed of ancestral SARS-CoV-2 and Omicron BA.4/5 (29 KTRs) or via natural infection with Omicron (38 BA.4/5, 31 BA.1/2), spike-specific T cells were quantified using Elispot and serum pseudovirus neutralizing activity was assessed against the ancestral Wuhan strain, BA.5 and XBB.1.5.

**Results:**

Compared to BA.4/5 vaccination, spike-specific T-cell responses and neutralization activity were higher up to six months post-Omicron infection and reached levels similar to healthy controls. Vaccinated KTRs showed modestly boosted neutralization activity against the Wuhan strain and BA.5, but not XBB.1.5. Baseline immunity correlated with immune responses three months post-vaccination and post-infection, indicating a predictive value for peak immune responses. Tixagevimab/Cilgavimab treatment was associated with robust neutralization of the Wuhan strain, but ineffective against XBB.1.5.

**Conclusion:**

The BA.4/5 vaccine improved neutralizing activity against the BA.4/5 variant, but not against the subsequently circulating XBB.1.5 variant in KTRs. Conversely, omicron infection boosted T cells and humoral responses more effectively, showing efficacy against XBB.1.5. These findings suggest that infection-induced immunity associates with greater protection than vaccination against future variants in KTRs.

## 1 Introduction

The SARS-CoV-2 pandemic posed a challenge to global health with the constant emergence of new variants. In 2022, the omicron variant emerged and became predominant worldwide, associated with a massive increase in infection rates (1, 2). Because high population immunity was achieved and the overall medical burden declined, measures to contain the spread of SARS-CoV-2 were reduced in many countries. Since then, multiple distinct Omicron subvariants have emerged with distinct antigenic properties (3). In June 2022 the BA.5 variant became predominant in Germany until January 2023, when it was replaced by new variants such as XBB.1.5. mRNA vaccines offer the possibility of rapid adaptation to new variants. Shortly after the approval of vaccines adapted to the Omicron variant BA.1 (Comirnaty Original/Omicron BA.1 and Spikevax Bivalent Original/Omicron BA.1), Comirnaty Original/Omicron BA.4-5 (adapted to Omicron BA.4/BA.5) was approved by the EMA in September 2022. These adapted bivalent vaccines were then recommended as booster vaccinations.

Kidney transplant recipients (KTRs) are among the most vulnerable groups regarding severe COVID-19, even after vaccination (4). This is mainly due to an abrogated B- and T-cell response after SARS-CoV-2 vaccination compared to healthy individuals (5–10). Data from the vaccine trials with adapted mRNA vaccines have demonstrated superior variant-specific immunity with adapted vaccines compared to the original vaccine (11, 12). Accordingly, booster vaccination with the BA.4/5 bivalent vaccines was recommended for KTRs by the German Standing Vaccination Commission in September 2022. Because a substantial subgroup of KTRs had not responded to prior booster vaccinations with detectable and sustained immune responses, we sought to determine how KTRs respond to an adapted vaccine and if variant-specific immunity against the currently circulating variant BA.5 as well as one of the subsequently circulating variants (XBB.1.5) was induced. Interestingly, coinciding with the start of the vaccination campaign, the incidence of SARS-CoV-2 Omicron infections increased, opening up the opportunity to directly compare SARS-CoV-2 specific immunity after BA.4/5 vaccination with immunity after SARS-CoV-2 BA.5 infections in KTRs. Because it is expected that KTRs will be exposed to SARS-CoV-2 at some point, the impact of the infection on SARS-CoV-2 immunity and specifically the immunity against future variants is of great interest.

Here, we studied a cohort of KTRs exposed to a 5^th^ SARS-CoV-2 antigen by either BA.4/5 vaccination or natural infection. SARS-CoV-2-specific T-cell and IgG levels were quantified and neutralizing activity against the original Wuhan strain, the BA.5 variant circulating at the time of the vaccination campaign and the XBB.1.5 variant following BA.5 was determined.

## 2 Methods

### 2.1 Study cohort

In this observational cohort study, KTRs who previously received four vaccine doses were included. The cohort is part of a previously described observational study (NCT04743947) of the Department of Nephrology, University Hospital Düsseldorf (7, 10, 13). We determined the immune response against SARS-CoV-2 before as well as 3 months and 6 months after their 5^th^ antigen contact. Inclusion criteria were: age ≥ 18 years, kidney transplantation before the first SARS-CoV-2 vaccine dose, four prior vaccinations with any licensed COVID-19 vaccine (from AstraZeneca, Johnson & Johnson, Moderna or BioNTech), being SARS-CoV-2 naïve before the 5^th^ antigen contact. KTRs were considered SARS-CoV-2 naïve when all of the following criteria applied: no documented positive SARS-CoV-2 test result, no self-reported positive SARS-CoV-2 antigen test, undetectable anti-nucleocapsid IgG and, when available, undetectable anti-nucleocapsid T cells before 5^th^ antigen contact. The study cohort was divided into 2 groups: KTRs vaccinated with the bivalent BA.4/5-adapted mRNA vaccine as the 5^th^ antigen contact (*Vac*); KTRs infected with SARS-CoV-2 as their 5th antigen contact (*Inf*). The group with infection was further subdivided into KTRs who were likely infected with BA.1 or BA.2 (*Inf BA.1/2)* and KTRs likely infected with BA.4 or BA.5 (*Inf BA.4/5)*. When no viral sequence data were available, the most prevalent variant at the time of infection was considered. According to public health reports of SARS-CoV-2 variants, from January to May 2022 the variants BA.1 and BA.2 and from June to December 2022 the variant BA.5 were predominant (**Supplementary Figure 1**). To evaluate the humoral immune response, infected KTRs were further divided into patients receiving or not receiving monoclonal antibodies. As a reference group, vaccinated, immunocompetent, non-transplanted individuals (age ≥ 18 years) were included who were infected with SARS-CoV-2 between June and December 2022. Due to the complexity of comorbidities in the KTR cohort, matching for pre-existing conditions was not performed. B- and T- cell immune responses were examined before, 3 and 6 months after the 5th antigen contact when possible (**Supplementary Table 1**). All KTRs and healthy controls gave written informed consent and the study was approved by the ethics committee of the Medical Faculty of the Heinrich-Heine-University Düsseldorf, Germany (ID 2020-1237 and 2021-1316).

### 2.2 Pseudovirus neutralization assay

Neutralization was determined using an in-house pseudovirus neutralization assay as previously described with minor modifications (14). This assay is based on a protocol for pseudotyping lentiviral particles (15). In brief, pseudoviruses were produced in HEK293T cells cultured at 37°C/5% CO_2_ by cotransfection of plasmids encoding the SARS-CoV-2 spike protein, HIV-1 Gag/Pol, HIV-1 Tat, HIV-1 Rev, and Luciferase-IRES-ZsGreen, respectively. Following a medium exchange, pseudovirus supernatants in cell culture medium (high glucose DMEM supplemented with 100 IU/ml penicillin, 2 mM L-glutamine, 1 mM sodium pyruvate, 100 µg/ml streptomycin (all Thermo Fisher) and 10% FBS (Sigma-Aldrich)) were harvested at 48 h and/or 72 h post-transfection, centrifuged, purified (0.45 mm filter) and stored at -80°C.

Heat-inactivated serum samples (45 min at 56°C) were tested in three-fold serial dilutions starting at 1:10. Following a 60 min co-incubation of serum samples and pseudoviruses at 37°C and 5% CO2, 293T-ACE2 cells were added. After two days, cells were lysed using luciferin/lysis buffer (0.3 mM ATP, 10 mM MgCl_2_, 17 mM IGEPAL CA-630, 0.5 mM coenzyme A (all Sigma-Aldrich), and 1 mM D-Luciferin (GoldBio) in Tris-HCL) and bioluminescence was determined using a microplate reader (Berthold). Average background relative light units (RLUs) of non-infected cells were subtracted, and serum 50% inhibitory dilutions (ID_50_s) were determined as the serum dilutions that resulted in a 50% reduction of RLUs compared to the average of virus-infected untreated controls cells using a non-linear fit model plotting an agonist versus normalized dose response curve with variable slope using the least squares fitting method in Prism (GraphPad).

Serum samples were analyzed at a starting dilution of 1:10 and in seven 3-fold serial dilutions (i.e., 1:10, 1:30, 1:90, 1:270, 1:810, 1:2430, 1:7290, 1:21870). Dilution series were performed in duplicate or triplicate and the geometric mean ID_50_ for each sample was calculated using the two individually determined ID_50_s closest to each other. The limits of quantification were defined by the lowest and highest serum dilutions tested. In case of neutralization above the upper limit of quantification (ULOQ) in one of the two ID_50_ values (ID_50_ >21.870), the sample was assigned an ID_50_ value of 21.870. If both ID_50_ values were above the ULOQ, the sample was assigned an ID50 of 43.740 (2x ULOQ). In case of neutralization below the lower limit of quantification (LLOQ) in one of the two values (ID_50_ <10), the sample was assigned an ID_50_ of 10. If both ID_50_ values were below the LLOQ, the sample was assigned an ID_50_ of 5 (1/2 x LLOQ).

### 2.3 Quantification of SARS-CoV-2 specific T cells

The SARS-CoV-2-specific T-cell response was quantified by a commercially available standardized Interferon gamma (IFNγ) release assay (T.SPOT COVID Test from Oxford Immunotec, UK) according to the manufacturer’s instructions. In brief, whole blood was collected and stored at room temperature until further processing within 24 hours. T-cell Xtend reagent (Oxford Immunotec, UK) was added and mononuclear cells were separated through density gradient centrifugation. Cells were counted and 2.5 x 10^5^ PBMCs were incubated for 20 hours with overlapping peptide pools covering the SARS-CoV-2 spike protein in the ELISPOT plate coated with anti-IFNγ. For each patient, analyses of negative (medium) and positive controls (phytohemagglutinine) were performed. On the next day, the plate was washed with PBS before IFNγ was stained. After the plate was dried, spots were counted using the BioReader 7000-E (Bio Sys, Germany). According to the manufacturer’s instructions <5 IFNγ spots (= spot forming units, SFU)/2.5 x 10^5^ PBMCs were considered negative. 5-7 SFU/2.5 x 10^5^ PBMCs were considered borderline and > 7 SFU/2.5 x 10^5^ PBMCs were considered positive.

### 2.4 Quantification of SARS-CoV-2-specific antibodies

Antibody levels against SARS-CoV-2 spike protein (anti-S antibodies) were quantified in serum using the SARS-CoV-2 QuantiVac ELISA (Euroimmun, Germany). According to the manufacturer’s instructions, results <25.6 BAU/ml were considered negative, values between 25.6-35.2 BAU/ml as borderline and values >35.2 BAU/ml as positive.

### 2.5 Statistics

Statistical analyses were performed with GraphPad Prism 9. Non-parametric tests were used for comparison of quantitative results between groups as indicated in the figure legends. P values <0.05 were considered as statistically significant.

## 3 Results

### 3.1 Study cohort

**Figure 1:**
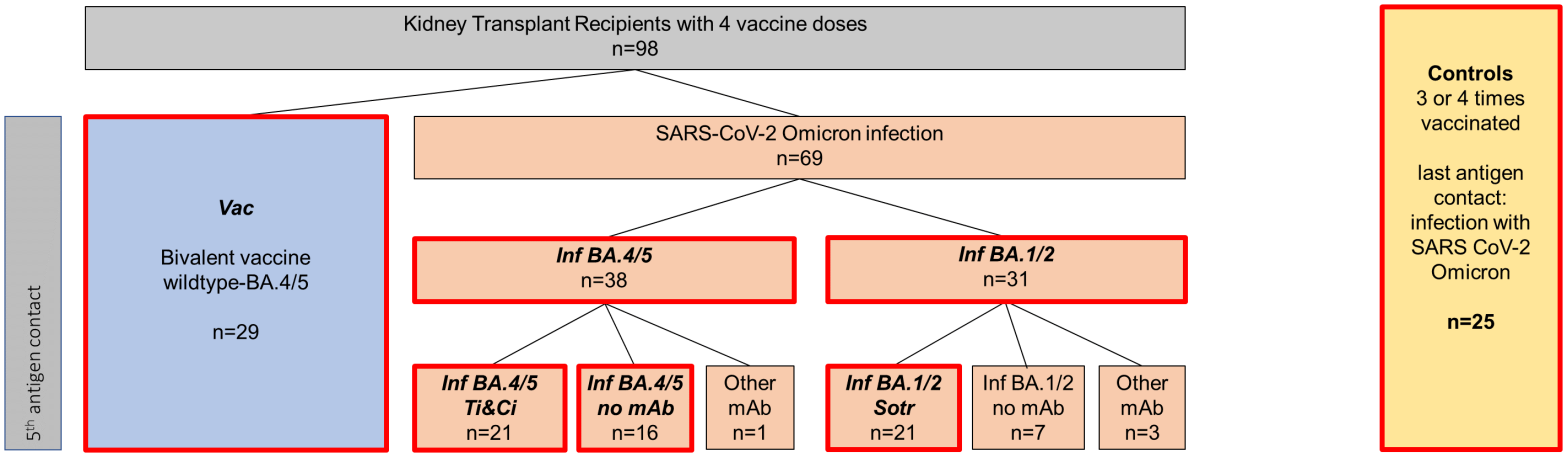
Classification of included patients into different cohorts. A total of 98 SARS-CoV-2 naive patients with four prior vaccinations were included. Of those, 29 patients received the wildtype/Omicron BA.4/5 bivalent vaccine as their fifth antigen exposure (highlighted in blue), referred to as “*Vac*” for “vaccinated kidney transplant recipients” throughout the manuscript. 69 patients were infected with SARS-CoV-2 Omicron as their fifth antigen exposure (represented in orange), with 38 putatively infected with Omicron BA.4/5 (*Inf BA.4/5*) and 31 putatively infected with Omicron BA.1/2 (*Inf BA.1/2*). 21 patients in the Inf BA.4/5 cohort received Tixagevimab/Calgevimab therapy, labeled “*Inf BA.4/5 Ti&Ci*” throughout the manuscript while 16 received no monoclonal antibody treatment and are classified as “ *Inf BA.4/5 no mAb*”. 21 BA.1/2 patients received Sotrovimab and are designated “ *Inf BA.1/2 Sotr*” Untreated BA.1/2 patients, as well as infected patients with other mAb than Tixagevimab/Cilgavimab or Sotrovimab were not analyzed separately due to small patient numbers. 25 healthy controls, who received 3-4 vaccine doses and who had a SARS-CoV-2 Omicron infection as their last antigen exposure, were included as a reference group.

A total of 102 KTRs with four documented vaccinations and no evidence of previous SARS-CoV-2 infection participated. Of these, 29 received the bivalent BA.4/5 vaccine as the 5^th^ antigen contact and were included in the vaccination cohort (*Vac*). 73 patients got infected with SARS-CoV-2 as their 5^th^ antigen contact. Samples from 35 patients were sequenced, resulting in 20 patients being assigned to the BA.1/2 cohort and 11 patients to the BA.4/5 cohort. 4 patients with other variants were excluded. Patients without SARS-CoV-2 sequencing data were assigned to the temporally predominant variant based on the period of their infection: 11 patients infected between January and May 2022 were assigned to the Omicron BA.1/BA.2 cohort (*Inf BA.1/2*), 27 patients infected between June and December 2022 were assigned to the Omicron BA.4/BA.5 cohort (*Inf BA.4/5*) (**Figure 1**). 24 of the 31 BA.1/2 infected patients and 22 of the 38 BA.4/5 infected patients received therapy with monoclonal antibodies (**Table 1**). None of the KTRs received Tixagevimab/Cilgavimab as pre-exposure prophylaxis. Regarding immunosuppression, 79% of the vaccinated cohort, 87% of the BA.1/2 infected cohort, and 68% of the BA.4/5 infected cohort received triple immunosuppression with mycophenolate mofetil (MMF), calcineurin inhibitors, and steroids. The remaining patients received other combinations. The MMF dosage and tacrolimus trough levels between the fourth and the fifth antigen exposure as a degree for the level of immunosuppression did not differ significantly between the three cohorts (**Supplementary Figure 2**). As infected patients presented with mild to moderate symptoms, the immunosuppressive treatment was not reduced during infection. In terms of vaccination history prior to the 5^th^ antigen contact, KTRs were also comparable: Only 14%, 10%, and 18% of patients in the *Vac*, *Inf BA.4/5*, and *Inf BA.1/2* cohorts had received a homologous vaccination scheme with mRNA-based vaccines only. The others had received at least one dose of a vector-based vaccine dose, either AstraZeneca or Johnson& Johnson (**Table 1**). 25 immunocompetent and non-transplanted individuals who had not yet received a bivalent vaccine but had a SARS-CoV-2 infection between June and December 2022 as their most recent antigen contact were included as controls, with 16 having received 3 vaccine doses before infection and 9 having been vaccinated 4 times.

**Table 1:** Base line characteristics.

	Vac n=29	Inf BA.1/BA.2 n=31	Inf BA.4/BA.5 n=38	Control group n= 25
Age, median (IQR), [y]	62, 17	63, 20.5	62, 11	50, 27
Sex (female)	11 (38%)	9 (29%)	12 (32%)	16 (64%)
Immunosuppressive treatment				
MMF + CNI + Steroids	23 (79%)	27 (87%)	26 (68%)	–
CNI + Steroids	2 (7%)	1 (3%)	3 (8%)	–
Other combinations	4 (14%)	3 (10%)	9 (24%)	–
Vaccination regime				
Dose 1				
AstraZeneca	2 (7%)	1 (3%)	4 (11%)	2 (8%)
Johnson&Johnson	0	0	0	1 (4%)
Comirnaty	27 (93%)	28 (90%)	32 (84%)	15 (60%)
Moderna	0	2 (6%)	2 (5%)	7 (28%)
Dose 2				
AstraZeneca	2 (7%)	0	1 (3%)	0
Johnson&Johnson	0	0	1 (3%)	1 (1%)
Comirnaty	27 (93%)	28 (90%)	34 (89%)	16 (64%)
Moderna	0	3 (10%)	2 (5%)	8 (32%)
Dose 3				
AstraZeneca	1 (3,5%)	0	2	0
Johnson&Johnson	1 (3,5%)	2 (6%)	0	0
Comirnaty	27 (93%)	29 (94%)	35 (92%)	19 (76%)
Moderna	0	0	1	6 (24%)
Dose 4				
AstraZeneca	0	0	0	0
Johnson&Johnson	0	0	0	0
Comirnaty	12 (41%)	11 (35%)	12 (32%)	8 (32%)
Moderna	17 (59%)	20 (65%)	26 (68%)	1 (4%)
Heterologous vaccination scheme	25 (86%)	28 (90%)	31 (82%)	22 (88%)
Therapy with monoclonal antibodies				
Casirivimab/Imdevimab	–	2 (6%)	0	–
Sotrovimab	–	21 (68%)	1 (3%)	–
Tixagevimab/Cilgavimab	–	1 (3%)	21 (55%)	–
No mAb	–	7 (23%)	16 (52%)	–
Antiviral treatment				
Molnupiravir	–	2 (6%)	17 (45%)	–
Remdesivir	–	19 (61%)	1 (3%)	–
Molnupiravir + Remdesivir	–	3 (10%)	7 (18%)	–
Other combinations	–	0	1 (3%)	–
none	–	7 (23%)	12 (32%)	–

### 3.2 SARS-CoV-2 Omicron infection but not bivalent SARS-CoV-2 BA.4/5 vaccination boosts the T cell immune response in KTRs

**Figure 2:**
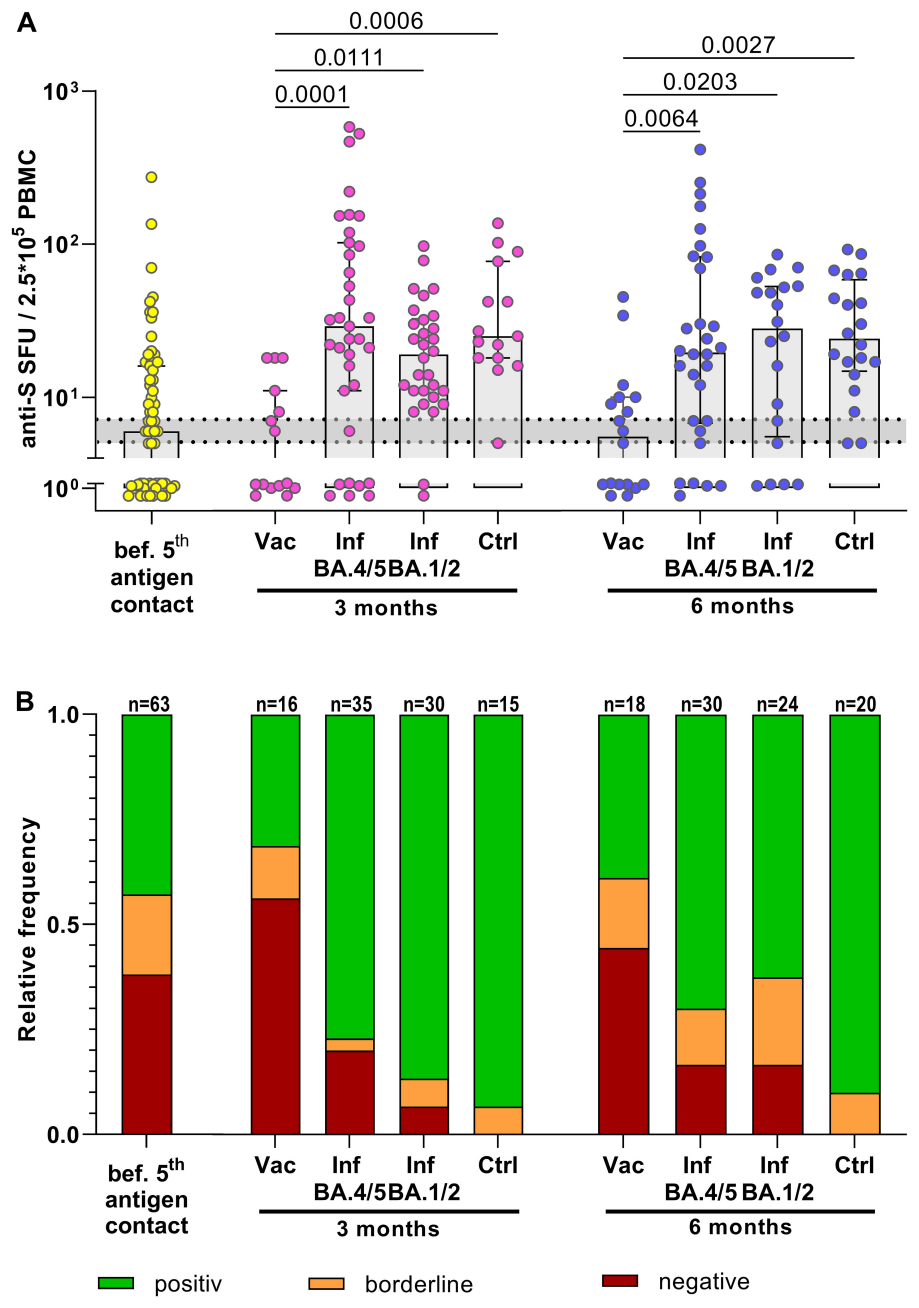
Boosted T-cell immune response in KTRs after SARS-CoV-2 Omicron infection but not after bivalent SARS-CoV-2 BA.4/5 vaccination. **(A)** Frequencies of anti-S specific T cells. Yellow: T-cell response in quadruple vaccinated KTRs before their 5th antigen contact. Rose/Blue: T-cell response in KTRs 3 months/6 months after their 5th antigen contact (BA.4/5 bivalent vaccine or BA.4/5 infection or BA.1/2 infection) compared to BA.4/5-infected healthy controls (Ctrl). An anti-S T-cell response >7 SFUs/2.5*10^5^ PBMCs was considered as positive, <5 SFUs/2.5*10^5^ PBMCs as negative. The range of borderline responses (5-7 SFUs/2.5*10^5^ PBMCs) is marked in grey. **(B)** Corresponding relative frequencies of a qualitative positive, borderline or negative anti-S specific T-cell response. For statistical analysis, Mann-Whitney test or Kruskal-Wallis-Test followed by Dunn’s *post-hoc* test was applied. Boxes and bars represent median and interquartile range.

The study compared the T-cell response to the SARS-CoV-2 spike protein in individuals who received the bivalent BA.4/5 vaccine with those who had contracted SARS-CoV-2 Omicron infection. Notably, KTRs with SARS-CoV-2 infection often received monoclonal antibody treatment (primarily Sotrovimab during the BA.1/2 wave and Tixagevimab/Cilgavimab during the BA.4/5 wave). The impact of monoclonal antibodies on SARS-CoV-2-specific T-cell immunity was therefore determined, revealing no significant difference in T-cell response between untreated infected KTRs and those treated with monoclonal antibodies (**Supplementary Figure 3**). Consequently, untreated and treated KTRs with SARS-CoV-2 infection were combined for T-cell response comparison. Following vaccination with the bivalent BA.4/5 vaccine, KTRs did not show improved T-cell responses at 3 and 6 months compared to pre-vaccination levels (**Figure 2**). Moreover, on a qualitative level, the proportion of KTRs with undetectable T-cell responses remained unchanged post-vaccination, with approximately half of KTRs still lacking detectable T-cell responses after the 5^th^ vaccine dose. This was in stark contrast to KTRs infected with SARS-CoV-2 BA.1/2 or Omicron BA.4/5, where the T-cell response significantly increased at 3 and 6 months post-infection compared to pre-infection levels. In contrast to vaccinated KTRs, the proportion of KTRs with undetectable T-cell responses decreased to 20% after BA.4/5 infection. Although the number of KTRs with undetectable T-cell responses was slightly higher 6 months after infection compared to healthy controls, the frequency of spike-specific T cells reached similar levels. Taken together, the induction of SARS-CoV-2 spike-specific T cells was substantially more robust after SARS-CoV-2 Omicron infection compared to SARS-CoV-2 BA.4/5 vaccination.

### 3.3 SARS-CoV-2 Omicron BA.4/5 infection induces stronger neutralization activity against BA.5 and XBB.1.5 than BA.4/5 bivalent vaccination in KTRs

**Figure 3:**
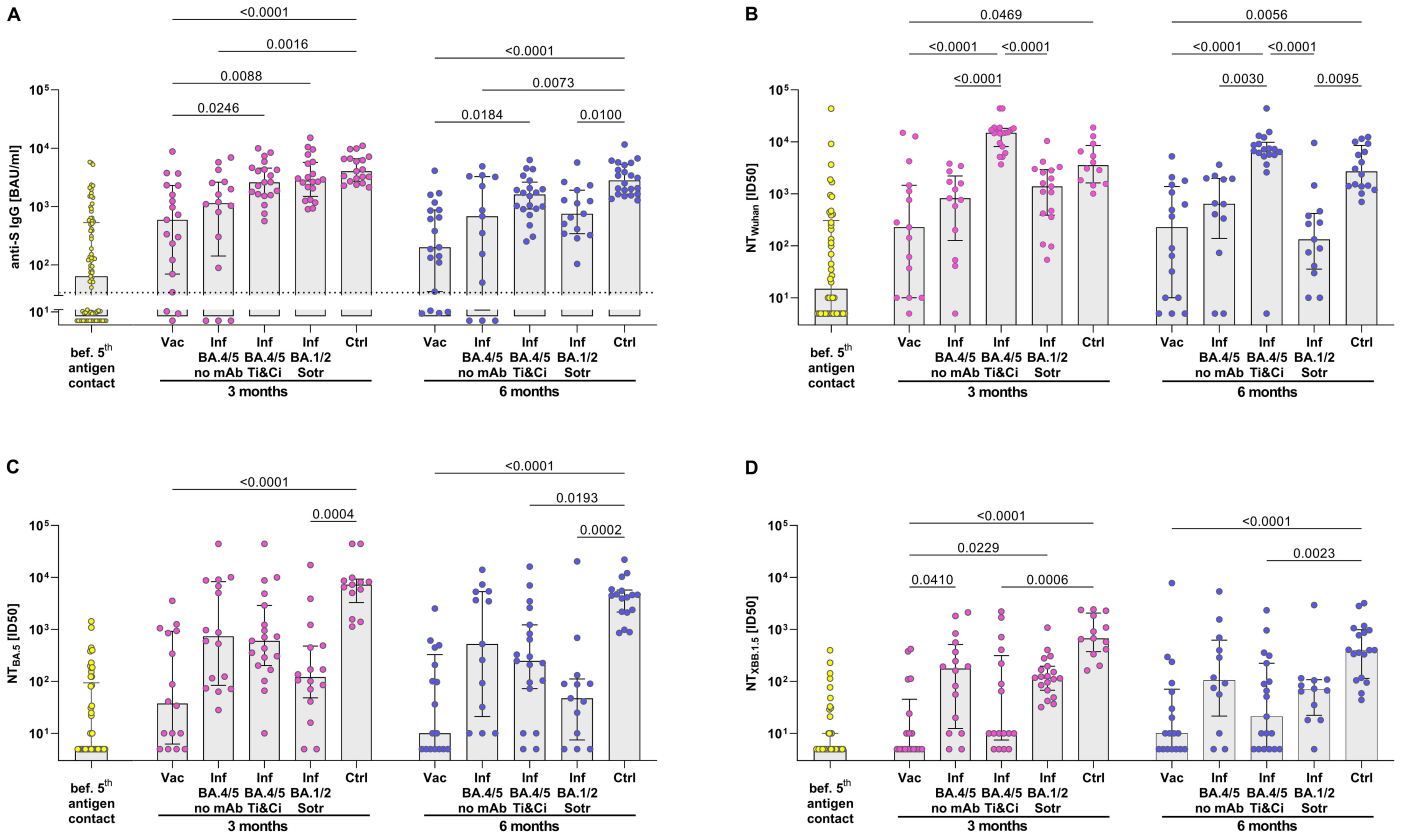
Stronger neutralization activity against BA.5 and XBB.1.5 in KTRs after SARS-CoV-2 Omicron BA.4/5 infection than after BA.4/5 bivalent vaccination. **(A)** Anti-S IgG Titers and **(B–D)** serum neutralization activity against Wuhan, Omicron BA.4/5 and Omicron XBB.1.5 pseudoviruses were determined. Yellow: B cell response in quadruple vaccinated KTRs before their 5th antigen contact. Rose/Blue: B cell response in KTRs 3 months/6 months after their 5th antigen: BA.4/5 bivalent vaccine (Vac) or BA.4/5 infection without treatment with monoclonal antibodies (Inf BA.4/5) or BA.4/5 infection receiving Tixagevimab/Cilgavimab (Inf BA.4/5 Ti&Ci) or BA.1/2 infection receiving Sotrovimab (Inf BA.1/2 Sotr) compared to BA.4/5 infected healthy controls (Ctrl). For statistical analysis, Kruskal-Wallis-Test followed by Dunn’s *post-hoc* test was applied. Boxes and bars represent median and interquartile range.

To compare SARS-CoV-2-specific antibodies and neutralization activity, SARS-CoV-2-infected KTRs treated with monoclonal antibodies were analyzed separately from untreated infected KTRs. This distinction was necessary because the assays cannot differentiate between the patient’s own humoral response and therapeutically applied antibodies as long as the viral strain in use is not completely resistant to the administered antibody. Three months after the 5^th^ vaccination, KTRs exhibited a 9.3-fold increase in the median anti-S IgG titer compared to pre-vaccination levels (**Figure 3**). However, this anti-S IgG titer (591 BAU/ml, IQR 2227 BAU/ml) remained lower than that in BA.4/5-infected KTRs (1143 BAU/ml, IQR 2478.7 BAU/ml) who had not received mAb therapy. As anticipated, SARS-CoV-2-infected KTRs treated with Tixagevimab/Cilgavimab or Sotrovimab showed higher levels of anti-S antibodies, similar to those observed after infection in healthy controls.

Neutralization activity post-vaccination and infection was assessed using pseudoviruses for the original Wuhan strain, the Omicron BA.5 variant, and the Omicron XBB.1.5 variant. At 3 months post-vaccination, there was a modest increase in neutralization activity against the Wuhan strain and the BA.5 variant, but no enhancement in neutralization of the XBB.1.5 variant. This suggests that the bivalent BA.4/5 vaccine boosted the antibody response against the original prototype and the BA.5 variant, but not against the subsequently circulating XBB.1.5 variant. This stands in contrast to KTRs with SARS-CoV-2 BA.4/5 infection, where neutralization was augmented against the Wuhan strain as well as both Omicron variants, including the XBB.1.5 variant. Consequently, at 3 and 6 months post-infection, neutralization activity against the three tested SARS-CoV-2 variants was higher in infected KTRs compared to vaccinated KTRs. Notably, the neutralization activity in infected KTRs almost reached levels comparable to healthy controls.

Treating infected KTRs with Tixagevimab/Cilgavimab or Sotrovimab also provided an opportunity to examine the long-term neutralization levels achieved with these monoclonal antibodies *in vivo*. At 3 and 6 months post-infection, KTRs treated with Tixagevimab/Cilgavimab exhibited significantly greater neutralization activity against the Wuhan strain compared to those treated with Sotrovimab. However, when it came to neutralizing the BA.5 and XBB.1.5 variants, there was no significant difference in activity between KTRs treated with Tixagevimab/Cilgavimab or Sotrovimab and those not treated with monoclonal antibodies. Interestingly, although not statistically significant, neutralization of the XBB.1.5 variant even tended to be lower in KTRs treated with Tixagevimab/Cilgavimab compared to untreated KTRs.

In summary, analyzing antibody levels in KTRs post-exposure to a 5^th^ antigen suggests overall higher neutralization activity after SARS-CoV-2 Omicron infection compared to bivalent BA.4/5 vaccination, including improved neutralization of the subsequently circulating XBB.1.5 variant.

### 3.4 Immunity prior to the 5^th^ antigen contact correlates with the level of immunity after vaccination or infection in KTRs

**Figure 4:**
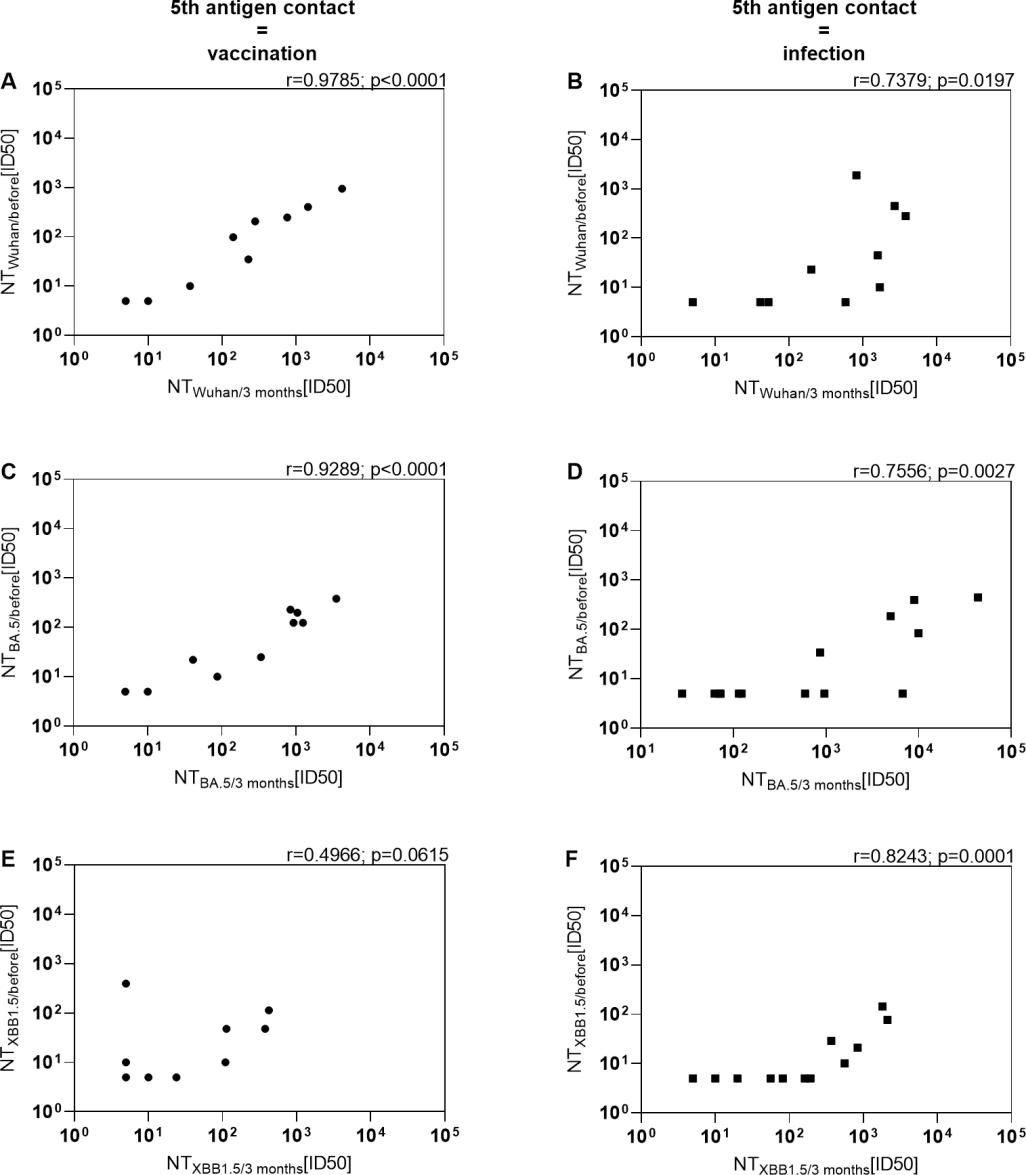
Positive correlation between level of immunity prior to the 5th antigen contact and level of immunity after vaccination or infection in KTRs. Serum neutralization activity against **(A, B)** Wuhan, **(C, D)** Omicron BA.5 and **(E, F)** Omicron XBB.1.5 before and 3 months after BA.4/5 bivalent vaccination or BA.4/5 infection were correlated. Left: KTRs with the bivalent BA.4/5 vaccination as their 5th antigen contact. Right: KTRs with an Omicron BA.4/5 infection as their 5th antigen contact without treatment with monoclonal antibodies.

We previously noted that among various factors, pre-existing immunity prior to vaccination predicts the immune response post-vaccination in KTRs (10). However, it remains uncertain whether this holds true in the context of infection. To explore this, we correlated neutralization activities against the Wuhan strain, the BA.5 variant, and the XBB.1.5 variant before and 3 months after the 5^th^ antigen exposure (**Figure 4**). Neutralization of the Wuhan strain and BA.5 variant before vaccination strongly correlated with neutralization 3 months after the bivalent BA.4/5 vaccine (r=0.9785; p<0.0001 and r=0.9289; p<0.0001). However, the correlation for neutralization of the XBB.1.5 variant was less robust and not significant (r=0.4966; p<0.0615), likely due to overall low levels of neutralization. Moreover, there was a significant correlation between neutralization of the Wuhan strain and the BA.5 variant before and 3 months after infection (r=0.7379; p=0.0197 and r=0.7556; p=0.0027), although the correlation coefficient was weaker compared to that after vaccination. Notably, in the context of KTR infection, neutralization of the XBB.1.5 variant before the 5^th^ antigen exposure strongly correlated with levels 3 months afterward. Taken together, these findings suggest that similar to immunity induced by vaccination, the level of humoral immune response prior to infection can serve as a predictor for the response level after infection in KTRs.

## 4 Discussion

Population immunity has substantially increased after vaccination campaigns have been implemented and was further promoted by natural SARS-CoV-2 infections, leading to a lower incidence of severe COVID-19 and significantly declined overall mortality (16–18). Protective measures such as contact bans or mandatory use of protective masks, predominantly implemented for protection of patient groups at high risk for severe COVID-19, were continuously lifted. At the same time, the immune evasive Omicron variant emerged and rapidly spread globally. Although the number of severe cases remained low, this resulted in unprecedented high SARS-CoV-2 infection rates as demonstrated in the highest peak concentrations of SARS-CoV-2 RNA in late 2023 since the beginning of waste water surveillance in Germany (19). It is expected that also vulnerable patient groups such as KTRs will be exposed to SARS-CoV-2 at some point. Accordingly, these groups continue to be a prime target of the vaccination strategy, in which vaccines are used that have been adapted to circulating SARS-CoV-2 variants.

In this study, the immune response to SARS-CoV-2 following vaccination with bivalent vaccines adapted to the BA.4/5 variants was directly compared with immunity post-SARS-CoV-2 Omicron infection in KTRs. The objective was to characterize immunity against presently circulating variants and potential future variants in immunocompromised KTRs. To facilitate a direct comparison, only KTRs who experienced their 5^th^ antigen encounter either through vaccination or SARS-CoV-2 infection were included. One notable strength of the study lies in its prospective nature, with analyses conducted on samples taken before vaccination and infection, respectively. This was made possible by utilizing an observational cohort study of KTRs that was initiated at the beginning of vaccination campaigns (7, 10, 13, 20), thereby ensuring comprehensive characterization of the immune response in all participants before the 5^th^ antigen contact.

A central finding was lack or only weak induction of T-cell responses by vaccination. This was in line with previous data on booster immunization with the standard vaccines in this cohort (10) and can now be extended to an adapted vaccine (21, 22). Of note, the peptides used for the ELISpot assay covered the SARS-CoV-2-spike protein of the Wuhan strain and were not adapted to the Omicron variant, however, it has been described that the differences in peptide sequences have no or only a minor effect on quantification of SARS-CoV-2 specific T cells (23–25). The data suggest that in KTRs activation and expansion of SARS-CoV-2 spike-specific T cells continues to be challenging even with mRNA vaccines, especially in those KTRs who also did not respond well to previous vaccines (26, 27). This is in contrast to SARS-CoV-2 Omicron infection, which led to a robust expansion of SARS-CoV-2 spike-specific T cells at 3 and 6 months post-infection. Similar results have also been observed in non-immunocompromised individuals, with stronger T-cell responses after breakthrough infection compared to fully vaccinated individuals (25).

Consistent with the literature, the 5^th^ vaccination with the bivalent BA.4/5 specific vaccine led to an increase in the anti-Spike IgG titers (28, 29). 3 months post-vaccination, anti-S IgG titers did not significantly differ between vaccinated and infected KTRs, underlying a quantitative robust B cell immune response after vaccination. In addition, 93% of the vaccinated cohort showed detectable neutralization activity against the Wuhan strain. In contrast, the neutralization activity was significantly reduced against the Omicron variant BA.5. which is in line with the previously reported immune evasive properties of this variant. After the first appearance of Omicron, continuous selection of immune evasive subvariants lead to the emergence and spread of XBB.1.5 in January 2023 after the vaccination campaigns had started with the adapted vaccines containing Omicron BA.4/5. The XBB.1.5 associated with a further decrease in neutralization activity (28, 30–32), which was confirmed here in our cohort of KTRs after the 5th vaccination. Collectively, our results suggest that a booster effect for virus neutralization was limited to the original Wuhan strain and to some extent to the BA.5 strain justifying adaption of the vaccine. However, in our cohort there was no significant booster effect on neutralizing activity on the serum level against the XBB.1.5 variant as a following emerging variant.

In turn, after BA.4/5 infection, peak neutralization activity was high and achieved levels 21.5-fold higher against BA.5 and 53-fold higher against XBB.1.5. compared to KTRs pos-vaccination. This strongly supports previous observations, that hybrid immunity induced by a combination of vaccination and infection is more robust and associates with broader neutralization capacity than vaccine-induced immunity (33–35). In KTRs this seems to be true for the humoral response as well as the T-cell response. Importantly, in this setting, hybrid immunity by vaccination and infection with the BA.5 variant also showed higher neutralization of the XBB.1.5 as the next following variant circulating in the population. As MMF may dose-dependently impair humoral immunity (20), lower immunosuppression in the infected cohort might theoretically account for their enhanced immune response. However, immunosuppression was not reduced in the infected cohort because infections were mild-to-moderate, and dosage of MMF and trough levels of Tacrolimus were comparable between vaccinated and infected cohorts. Additionally, absolute anti-Spike IgG titers did not differ significantly between vaccinated and mAb-untreated infected KTRs. Thus, different levels of immunosuppression is unlikely to account for the observed differences in humoral immune responses between KTR groups. Although SARS-CoV-2 infections in vaccinated KTRs unfortunately still come with the risk of severe disease, they seem inevitable when the infection pressure in the population is high as has been observed during the season 2022/23. In these cases, infection associated immunity may strongly contribute to protection and also promotes immunity against future variants, which seems difficult to achieve in immunosuppressed KTRs by vaccination only.

Finally, the application of monoclonal antibodies as SARS-CoV-2 treatment in many KTRs opened up the opportunity to study the neutralizing levels achieved *in vivo* against the different variants. Although the mAb combination Tixagevimab/Cilgavimab was clearly associated with a high neutralizing activity against the Wuhan strain in serum over a period of at least 6 months, there was only limited activity against the BA.5 variant and no activity against the XBB.1.5 variant. This was expected as Tixagevimab/Cilgavimab has been described to be not effective against XBB.1.5 variant in healthy patients treated with these mAbs as pre-exposure prophylaxis and because XBB.1.5 is resistant to Tixagevimab/Cilgavimab *in vitro* (36, 37). Interestingly, 10 of 17 Tixagevimab/Cilgavimab-treated KTRs (59%) showed no relevant neutralizing activity (<=10) against XBB.1.5 compared to 24% in the mAb-untreated cohort (4 of 17). Although not conclusive, a negative effect of Tixagevimab/Cilgavimab on the induction of novel variant-specific antibodies by the host should be considered. Recent data suggest, that the administration of monoclonal antibodies has an effect on memory B cell selection (38, 39). Nevertheless, the possible negative consequences of therapeutically applied antibodies lacking activity against the relevant variant on the development of an autologous humoral response need further investigation. An alternative explanation for weaker neutralization of XBB.1.5 in the Tixagevimab/Cilgavimab-treated cohort compared to mAb-untreated KTRs may be a potential selection bias, as only those KTRs who sought medical attention at the hospital received treatment with Tixagevimab/Cilgavimab. A possible negative impact on neutralization activity was not observed for Sotrovimab-treated BA.1/2 infected KTRs. This is in line with existing data, reporting some residual neutralization activity against XBB.1.5 in Sotrovimab-treated patients in contrast to Tixagevimab/Cilgavimab treated patients (40, 41). Nevertheless, in the context of continuous evolution of subsequent variants with even higher immune evasive properties, both Tixagevimab/Cilgavimab and Sotrovimab are not generally recommended any longer (42).

There are few limitations to the study. Data were analysed pseudo longitudinally instead of longitudinally, due to the observational nature of this study, which integrated patients from routine clinical visits without consistent sampling at all time points. This led to incomplete data across cohorts, especially in the BA.1/2-infected group, where 25 of 31 KTRs missed evaluations between the fourth and fifth antigen exposures. At that stage, all KTRs had received four vaccinations and were SARS-CoV-2 naive, ensuring equivalent antigen exposure, with cohort differentiation only occurring at the fifth exposure. As we have almost complete data for vaccinated and BA.4/5-infected cohorts at this time, we believe the missing BA.1/2 data did not significantly impact our findings. Considering the comparability of the cohorts, it is notable that the control group was slightly younger than the KTRs (p = 0.011). However, the control group was included as a reference to contextualize the immune response of the KTRs. No conclusions were drawn based on direct comparisons between healthy controls and KTRs. Hence, the age difference of the control group was not considered to be a problem when immune responses of vaccinated KTRs and infected KTRs were compared. Furthermore, the higher proportion of women in the control group should be noted, as sex-specific differences in the immune response exist, which are particularly complex and thus are not further discussed here (43). The exact vaccination histories regarding the specific vaccines of the included patients are heterogeneous. However, this represents the real-world scenario, as the choice of vaccine was influenced by external factors such as vaccine availability, which changed over time with the approval of new vaccines. Here, 86% of the vaccinated KTRs, 90% of the BA.1/2 infected KTRs, and 82% of the BA.4/5 infected KTRs had a prior vaccination history with at least one dose of a vector-based vaccine. Thus, the patient groups appear roughly comparable in this regard. Furthermore, the exact SARS-CoV-2 genotype was determined only for the minority of the infected patients. Therefore, the most likely genotype was inferred from data on the circulating variants in Germany published by the public health authorities. The inclusion of infected KTRs as BA.4/5 or BA.1/2 infected KTRs was based on the time of infection and the predominant variant at that time (BA.4/5 between June and December 2022 and BA.1/2 between January to May 2022). This represents the real-life scenario when therapeutic decisions are based on the predominant variants. Because the distribution of variants was overall rather homogenous during that time, we believe that inference of the genotype base on epidemiological data is a reasonable proxy. Importantly immunity induced by infections with the XBB.1.5 variant can be excluded, because this variant was not circulating in Germany during that time. In the infected cohort, variations in treatment approaches, such as the use of different mAbs, present an additional limitation to the study, as they may have influenced the humoral immune response differently. Treatment data with mAbs was available for all infected patients, allowing us to compare groups according to the monoclonal antibody therapy. Importantly, we were also able to include an untreated BA.4/5 infected cohort. Therefore, we believe this limitation does not compromise the overall validity of our data.

## Data Availability

The original contributions presented in the study are included in the article/**Supplementary Material**. Further inquiries can be directed to the corresponding author/s.
