# Supplementary figures and images for: Comparison of immune responses to SARS-CoV-2 spike following Omicron infection or Omicron BA.4/5 vaccination in kidney transplant recipients
Source: Front Immunol. 2025 Jan 14;15:1476294. doi: 10.3389/fimmu.2024.1476294 (PMC11772199; doi:10.3389/fimmu.2024.1476294)

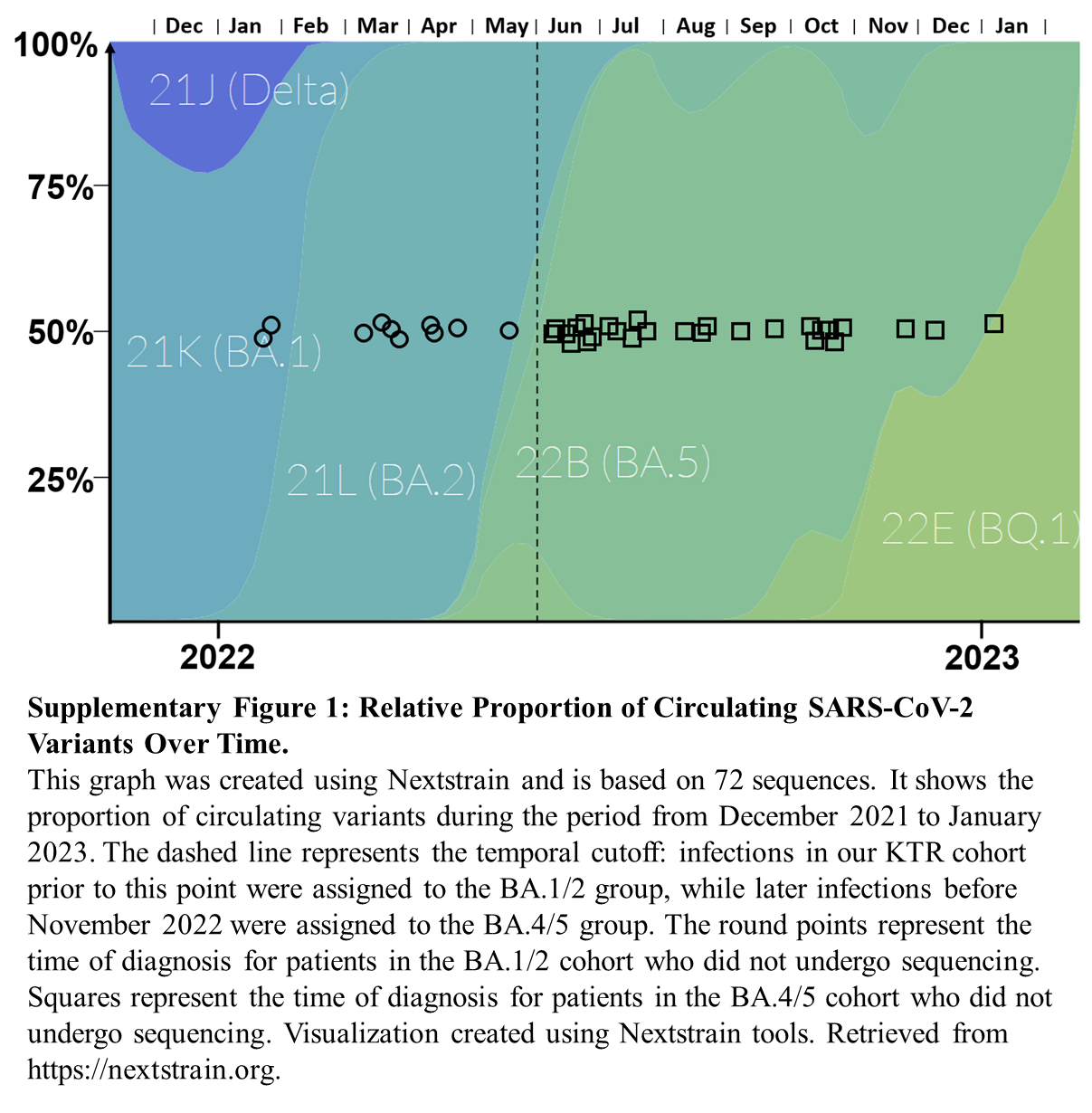

Supplement: Supplementary file 1 [file Image1.tif]

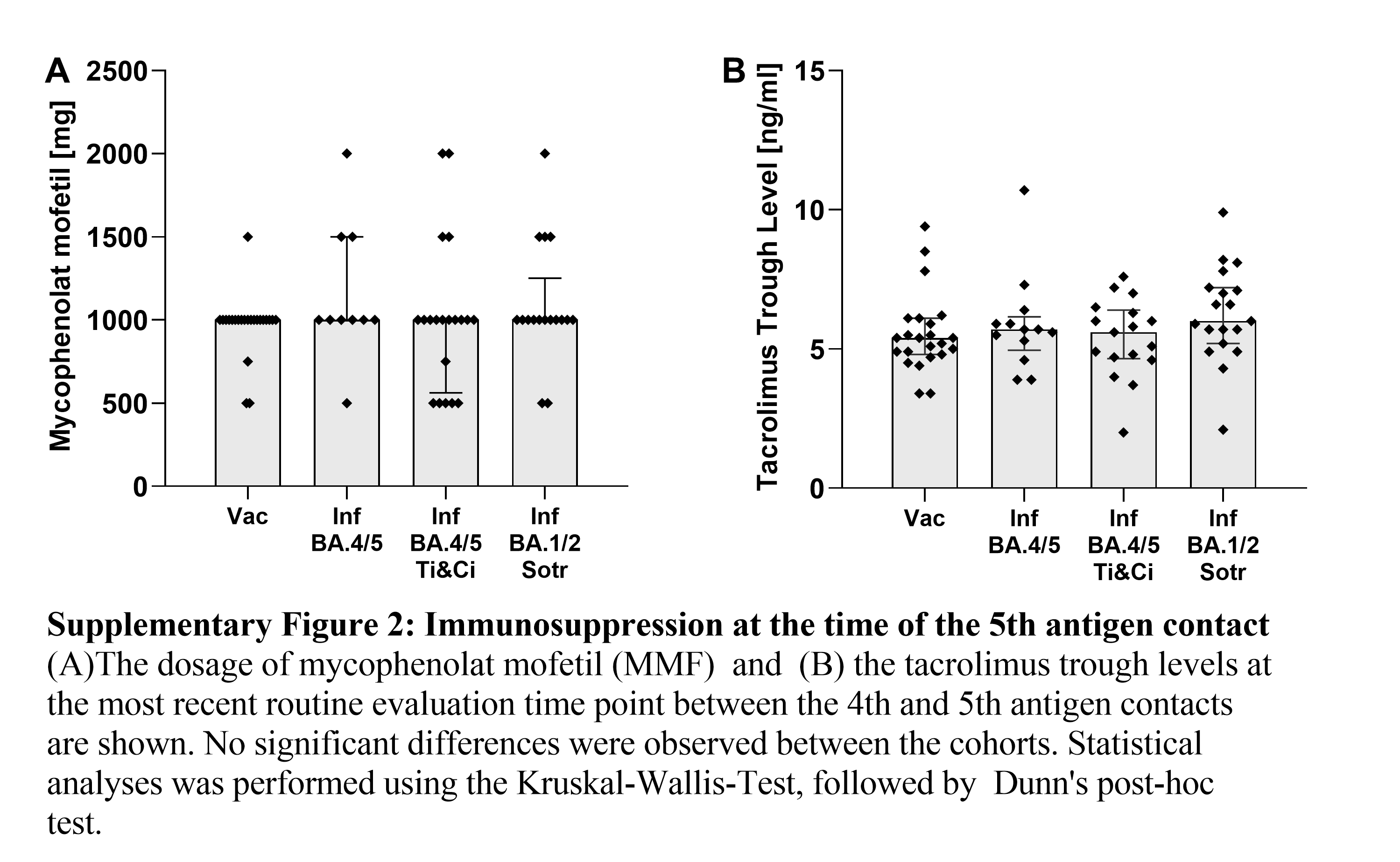

Supplement: Supplementary file 2 [file Image2.tif]

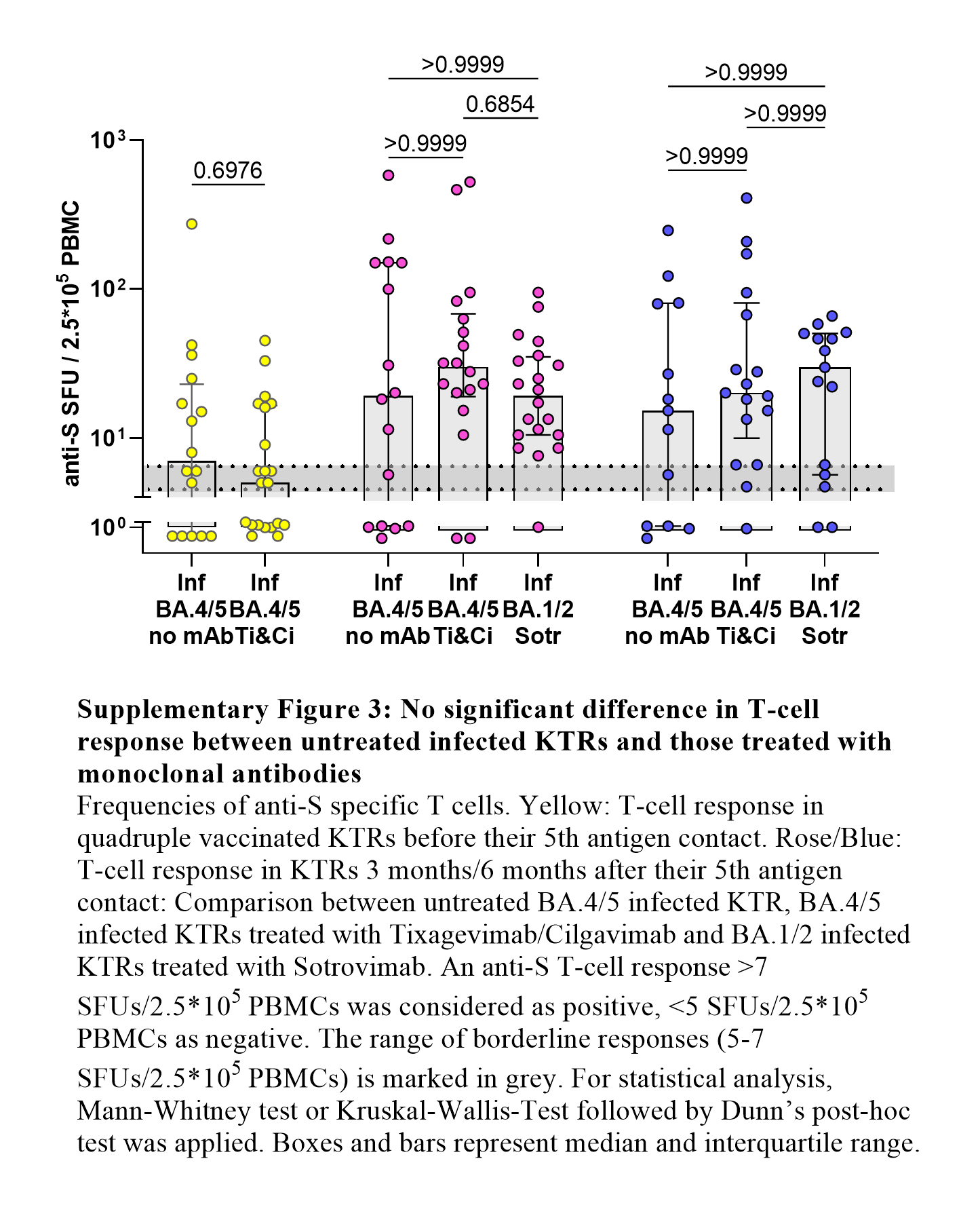

Supplement: Supplementary file 3 [file Image3.tif]
