# Supplementary material for: Comparison of immune responses to SARS-CoV-2 spike following Omicron infection or Omicron BA.4/5 vaccination in kidney transplant recipients
Source: Front Immunol. 2025 Jan 14;15:1476294. doi: 10.3389/fimmu.2024.1476294 (PMC11772199; doi:10.3389/fimmu.2024.1476294)
Supplement: Supplementary file 4 [file Table1.docx]

**SUPPLEMENTS**

| **Supplementary Table 1: Number of examinations carried out per cohort and time point** | | | |
| --- | --- | --- | --- |
|  | **analysis prior to 5^th^ antigen contact** | **3 months after 5^th^ antigen contact** | **6 months after 5^th^ antigen contact** |
| **Vaccination n=29** | | | |
| n=17 | x | x | x |
| n=1 | x | x |  |
| n=1 | x |  | x |
| n=9 | x |  |  |
| n=1 |  |  | x |
| **Infection BA.1/2 Jan-June 2022 n=31** | | | |
| n=5 | x | x | x |
| n=6 |  | x |  |
| n=1 | x | x |  |
| n=18 |  | x | x |
| n=1 |  |  | x |
| **Infection BA.4/5 June-Dec 2022 n=38** | | | |
| n=29 | x | x | x |
| n=2 | x |  |  |
| n=6 | x | x |  |
| n=1 |  |  | x |
| **Healthy controls n=25** | | | |
| n=11 |  | x | x |
| n=6 |  | x |  |
| n=8 |  |  | x |
